# A Bioactive Hydrogel and 3D Printed Polycaprolactone System for Bone Tissue Engineering

**DOI:** 10.3390/gels3030026

**Published:** 2017-07-06

**Authors:** Ivan Hernandez, Alok Kumar, Binata Joddar

**Affiliations:** 1 Inspired Materials & Stem-Cell Based Tissue Engineering Laboratory (IMSTEL), Department of Metallurgical, Materials and Biomedical Engineering, University of Texas at El Paso, El Paso, TX 79968, USA; ihernandez38@miners.utep.edu; 2 Border Biomedical Research Center, University of Texas at El Paso, El Paso, TX 79968, USA; bjoddar@utep.edu

**Keywords:** 3D printing, polycaprolactone (PCL), hydroxyapatite, hydrogel, bone defect

## Abstract

In this study, a hybrid system consisting of 3D printed polycaprolactone (PCL) filled with hydrogel was developed as an application for reconstruction of long bone defects, which are innately difficult to repair due to large missing segments of bone. A 3D printed gyroid scaffold of PCL allowed a larger amount of hydrogel to be loaded within the scaffolds as compared to 3D printed mesh and honeycomb scaffolds of similar volumes and strut thicknesses. The hydrogel was a mixture of alginate, gelatin, and nano-hydroxyapatite, infiltrated with human mesenchymal stem cells (hMSC) to enhance the osteoconductivity and biocompatibility of the system. Adhesion and viability of hMSC in the PCL/hydrogel system confirmed its cytocompatibility. Biomineralization tests in simulated body fluid (SBF) showed the nucleation and growth of apatite crystals, which confirmed the bioactivity of the PCL/hydrogel system. Moreover, dissolution studies, in SBF revealed a sustained dissolution of the hydrogel with time. Overall, the present study provides a new approach in bone tissue engineering to repair bone defects with a bioactive hybrid system consisting of a polymeric scaffold, hydrogel, and hMSC.

## 1. Introduction

Traditionally, bone fractures and defects created due to injury or disease are treated by temporary and/or permanent implants [1]. However, inadequate bone growth leads to non-union of newly formed bone in such circumstances [2,3]. Therefore, a major concern in repairing bone defects is absence of a suitable implant to accelerate bone regeneration and induce bone union. Alternatively, in bone-tissue engineering, biomaterials alone or in combination with suitable biological and chemical factors are used to restore the functionality of injured bone tissue [4]. In this context, implantation of cell-seeded scaffold constructs have been used to enhance bone regeneration [5,6]. Furthermore, presence of a bioactive biomaterial, such as autologous bone harvested from the patient’s own body or osteogenic supplements can be helpful in bone formation [7,8]. Therefore, in the past, several methods have been developed for delivering osteoblasts (bone forming cells) and osteogenic growth factors at defect sites [8,9,10]. Concurrently, three dimensional (3D) printing methods have been used to create uniquely designed scaffolds for faster recovery from bone injuries [11]. 3D printed porous scaffolds with interconnected pores should allow the formation of vascularized tissue, which is required to supply nutrients and oxygen to growing cells inside the pores [12]. Unlike very expensive additive manufacturing methods such as electron beam melting (EBM) and selective laser sintering (SLS) used to fabricate high strength scaffolds, 3D printing methods such as fused deposition modeling (FDM) can alternatively be used to fabricate scaffolds at much lower costs [11].

Among various characteristics required for reconstruction of bone defects, a patient-specific design mimicking the fractured bone, with an ability to promote bone ingrowth and healing is required for faster recovery from bone-injuries. Therefore, in this study, we developed a hybrid system of a 3D printed scaffold of polycaprolactone (PCL) and bioactive hydrogel infiltrated with human mesenchymal stem cells (hMSC). Our overall objective was to develop an on-demand method to provide a support system for application in segmental bone defect restoration with a custom-made PCL scaffold, which would also deliver a bioactive hydrogel and hMSC for induction of bone growth. Furthermore, this PCL/hydrogel system would not be cytotoxic and would support bone repair as it contained hMSC. The novelty in this study is the simultaneous application of both a hydrogel (with hMSC) and a 3D printed PCL scaffold to make a hybrid system with bioactive properties and capability to support and maintain the structural integrity of bone, during repair and regeneration.

## 2. Results

### 2.1. Hydrogel Preparation

The hydrogel was prepared by the crosslinking of an alginate and gelatin mixture with 1-ethyl-3-(3-dimethylaminopropyl) carbodiimide (EDC) and *N*-hydroxy-succinimide (NHS), followed by calcium chloride (CaCl_2_). During hydrogel synthesis, EDC was used to activate the carboxyl groups of alginate to form active ester groups, followed by NHS bonding with alginate due to replacement of EDC to improve the efficiency of amine reaction [13,14]. To synthesize the pre-hydrogel (Figure S1), NHS activated carboxylic group of alginate was linked with the primary amine of gelatin by replacing NHS. Addition of CaCl_2_ led to the ionic interactions of α-l-guluronic acid (G-block) of alginate in the pre-hydrogel to form a stable crosslinked hydrogel. Cross-linking with CaCl_2_ was done to enhance the retention of the hydrogel within the PCL scaffold structure (Figure S2).

### 2.2. Rapid Fabrication of the 3D Printed PCL Scaffold

Using FDM technology, primarily, a cylindrical-shaped gyroid PCL scaffold (height: 5 mm, diameter: 15 mm) was printed within 10 min (Figure S3). Mesh and honeycomb structures of similar dimensions were also printed to compare the hydrogel retention capacity of scaffolds.

### 2.3. Hydrogel Retention Capacity of the PCL Scaffolds

**Figure 1:**
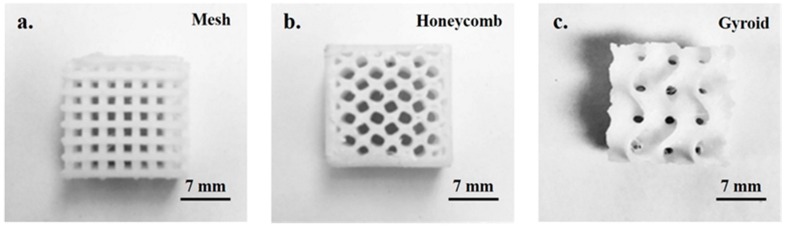
Digital pictures of 3D printed mesh (**a**), honeycomb (**b**), and gyroid (**c**) structures of identical dimensions.

As shown in Figure 1, three different 3D printed structures: mesh, honeycomb, and gyroid of identical dimensions were filled with hydrogel to compare the irrespective gel loading capabilities. Results showed an average of 1.25 ± 0.04 g, 0.82 ± 0.04 g and 0.46 ± 0.04 g of hydrogel loaded in the gyroid, mesh, and honeycomb structures, respectively. The amount of hydrogel loaded within the gyroid was about ~35% greater compared to mesh and ~63% greater compared to honeycomb, respectively. Since the 3D PCL scaffold took less than 10 min to print, this composite PCL/hydrogel implant can be applied to an in vivo application within a desired and short time frame if needed. Furthermore, we noted a higher amount of hydrogel retention in gyroid structure than in mesh and honeycomb structures. Therefore, only gyroid structure was used for further study.

### 2.4. Microstructure Imaging and Characterization of Phases in the PCL Scaffold and Hydrogel System

**Figure 2:**
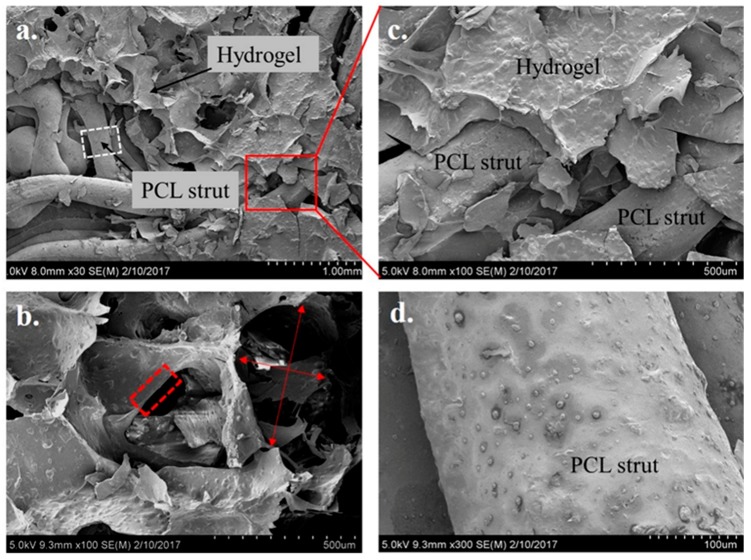
Scanning electron microscope (SEM) images of freeze-dried polycaprolactone (PCL)-gel samples (**a**). A high magnification image confirmed the highly porous nature of the hydrogel with interconnected pores. The pore shape and pore wall thickness are marked with a cross-arrow and a rectangular box, respectively (**b**). A magnified image of region marked with rectangular box in (**a**) showed complete adherence of hydrogel on the scaffold, which is expected to provide a bioactive coating to the otherwise bioinert surface of PCL (**c**). The PCL scaffold was characterized by surface micro-roughness and non-homogeneity (**d**).

**Figure 3:**
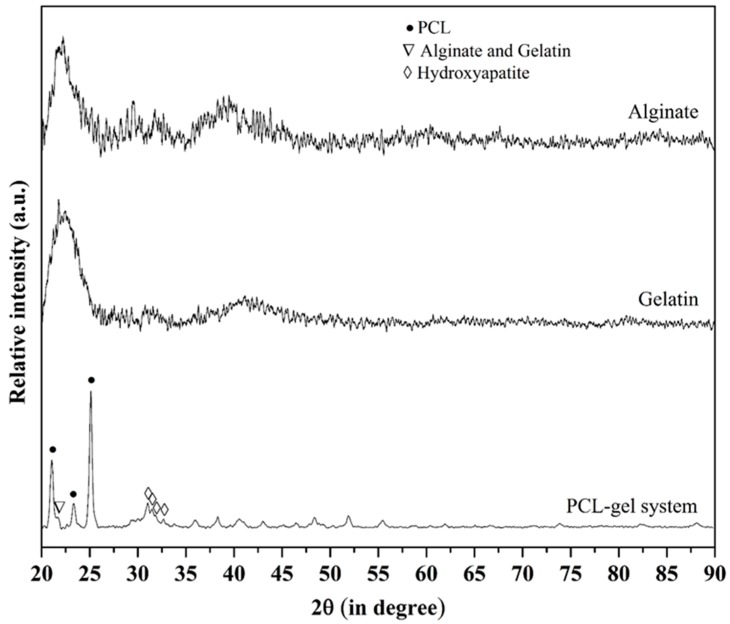
A comparison of X-ray diffraction (XRD) data of hybrid PCL/hydrogel scaffolds with alginate, and gelatin confirmed the presence of semi-crystalline phases of alginate and gelatin in the hydrogel loaded in the PCL scaffold (∇). The diffraction data also confirmed the presence of PCL (•) and hydroxyapatite (HA) (◊) in its monolithic phase.

Scanning electron microscope (SEM) images revealed a highly porous structure of the hydrogel with an average pore size of 399.22 ± 22.03 µm (Figure 2). The PCL scaffold (gyroid) was characterized by an average strut diameter of 320.17 ± 3.47 µm. X-ray diffraction (XRD) data showed the presence of signatory diffraction peaks of hydroxyapatite (HA), alginate and gelatin in the hydrogel (Figure 3). The sharp narrow peaks of HA and PCL confirmed the crystallinity of the nano-HA and PCL.

### 2.5. Sustained Dissolution of Hydrogel in Simulated Body Fluid (SBF)

**Figure 4:**
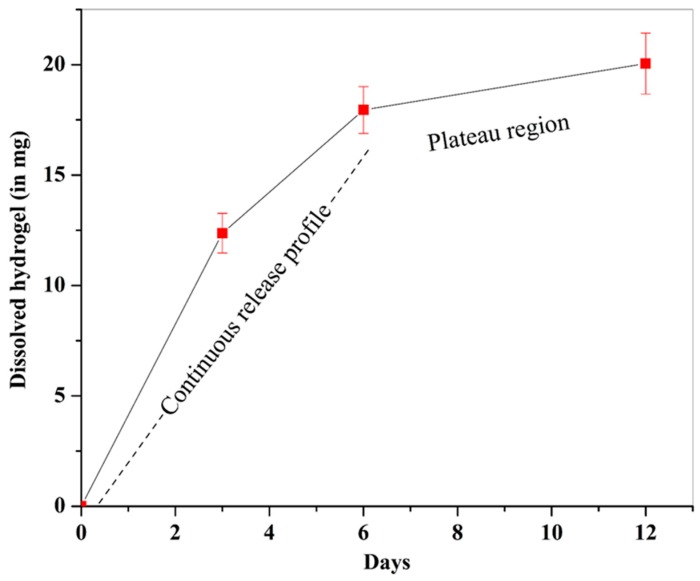
The dissolution study carried out in simulated body fluid (SBF) for 3, 6, and 12 days showed the continuous dissolution of hydrogel with time, with decrease in dissolution rate after 3 days. A plateau region after 6 days can either be associated with significant decrease in degradation rate of hydrogel or predominant apatite deposition from the SBF (see Figure 5).

Constant visual monitoring of the samples during and after the completion of the dissolution study in SBF showed a uniform dissolution of hydrogel during the 12 days of test period. After the test, optical density of the spent SBF was measured to estimate the amount of dissolved hydrogel (Figure 4). This measured optical density was converted to actual amounts of hydrogel dissolved per unit volume of spent SBF, based on a standard curve (Figure S4). Results revealed a continuous dissolution profile for hydrogel from day 1–6 of the study. An average of 12.37 ± 0.90 mg hydrogel dissolved in the first three days, followed by 5.58 ± 0.16 mg hydrogel dissolution in next 3 days. After 6 days, a decrease in the rate of dissolution was noted with only 2.1 ± 0.32 mg hydrogel found dissolved in the next 6 days.

### 2.6. Apatite Formation Ability of the PCL/Gel System

**Figure 5:**
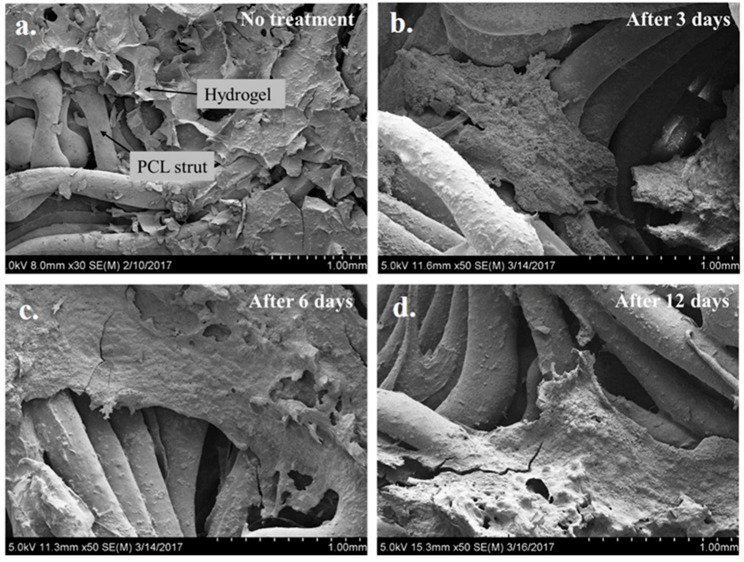
Low magnification SEM images of freeze-dried PCL-gel samples without SBF (**a**) and with SBF treatment for 3 (**b**), 6 (**c**), and 12 days (**d**). The SBF treated samples showed homogenous apatite layer over the hydrogel as well as PCL struts with an increasing amount of apatite deposition with time. A crack in apatite layer in (**c**,**d**) is due the strain generated due to drying of the samples.

**Figure 6:**
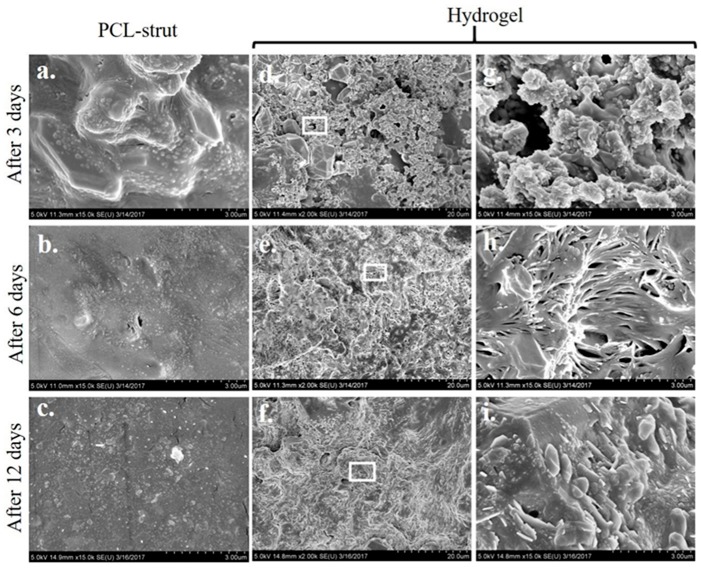
High magnification SEM images of freeze-dried PCL/ hydrogel samples after 3 (**a**,**d**,**g**), 6 (**b**,**e**,**h**), and 12 days (**c**,**f**,**i**) of immersion in SBF. The (**g**), (**h**), and (**i**) are the magnified images of regions marked in micrographs (**d**), (**e**), and (**f**), respectively. Results showed the deposition of apatite on both PCL as well hydrogel (**a**,**d**) in the initial period (3 days) of SBF immersion. A lower amount of apatite on PCL struts than hydrogel after 6 and 12 days may be due to the dissolution of deposited apatite from PCL. Scale bar for (**a**–**c**,**g**–**i**) is 3 μm and for (**d**–**f**) 20 μm.

The in vitro apatite formation ability of a biomaterial can be correlated to its in vivo bone-bonding ability [15]. In this context, samples used in the dissolution study in SBF were further used to study the formation and dissolution of apatite within these PCL-gel samples. SEM images showed a uniform deposition of apatite with higher amounts deposited within the hydrogel as compared to PCL scaffolds (Figure 5). After 3 days of immersion in SBF, deposition of apatite crystals was noted. After 6 and 12 days (Figure 5c,d, respectively), a thick layer of apatite was noted due to the continuous deposition of apatite. A strain-induced crack, resulting from drying of the samples was used to estimate the apatite layer thickness after 12 days, which revealed a thickness of 15.62 ± 0.51 μm (Figure S5). Higher magnification SEM micrographs showed a higher amount of deposited apatite after 3 days on PCL struts as compared to after 6 and 12 days (Figure 6a–c). In contrast, as shown in Figure 6d–f, more apatite formation with time was observed with a relatively denser apatite layer noted on day 12. Higher magnification images (Figure 6g–i) showed a change in morphology of the deposited apatite with time. After 3 days, a globular morphology of apatite was noted on the hydrogel, which changed to acicular morphology on day 6. After 12 days, a denser apatite layer was observed with rod-like fine spherical-shaped apatite crystals.

In summary, time dependent apatite formation and stabilization was noted within the hydrogel with a higher amount of apatite on day 12 as compared to days 3 and 6. However, a smaller amount of apatite was formed on the PCL surface which was reduced with time due to noticeable amounts of dissolution of apatite layer from the PCL surfaces.

### 2.7. Cytocompatibility of the PCL/Gel System

**Figure 7:**
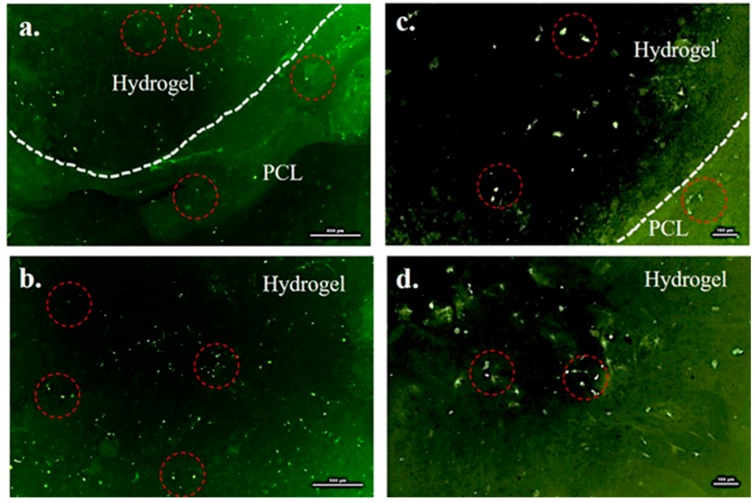
Representative fluorescence images of PCL-gel samples seeded with pre-stained human mesenchymal stem cells showed the presence of cells (green) in the hydrogel (**a**,**b**) as well as on the PCL struts (**a**). The white-colored broken line shows the boundary between the PCL scaffold and hydrogel. The cells are marked with red circles within both the hydrogel and scaffold areas. Images (**c**,**d**) are the magnified images of micrographs (**a**,**b**), respectively. Scale bar for (**a**,**b**) is 500 μm and for (**c**,**d**) is 100 μm.

Figure 7a showed the presence of viable pre-stained hMSC in the entire system, although there were more cells in the hydrogel than on the scaffold strut (Figure 7b). Since PCL is bioinert in nature and does not support cell adhesion [16], a bioactive hydrogel infiltrated with hMSC was loaded within the pores of the scaffold. Due to the bioactive property of the hydrogel, we expected a higher number of viable cells to be retained within the hydrogel. Figure 7c showed a large number of cells with elliptical-shaped morphology (Figure 7d). Control samples did not fluoresce at all (Figure S6), confirming the presence of viable hMSC in the samples imaged and reported in this study.

## 3. Discussion

Hydrogels have been widely used for most tissue engineering applications [17]. Specifically, hydrogel allows higher cell encapsulation and therefore, delivery of a greater concentration of cells at the site of implantation/defect, which in turn could accelerate the regeneration of the damaged tissue [18,19]. However, they possess weak mechanical properties that can be optimized by functionalization or crosslinking [20,21], or by serving as a bioactive filler material within a bioinert scaffold, which by itself does not interact with the body [22]. However, such a scaffold can still be structurally capable of supporting cell-growth, cell-proliferation, and vascularization [23]. The idea of using a supporting scaffold and a bioactive filler material with cells was based on the work by Gugala et al. [7], in which a bioactive graft material and a porous support structure was used to restore a bone defect in vivo. No healing was noted in the absence of any support material. But, a partial healing was found in case of perforated polylactic acid sheet used as a scaffold [7]. In contrast to this, complete healing was achieved when this scaffold was filled with autogenous cancellous bone [7]. In summary, this study emphasized the importance of both a support scaffold and a bioactive material to promote bone regeneration [7]. Considering the importance of the role played by the presence of osteogenic material in new bone formation, in the present study a novel approach was explored to incorporate hMSC and HA in an alginate and gelatin-based hydrogel and filled within a 3D printed PCL scaffold. Multipotent stromal hMSC were added to improve the osteoconductive properties of the hydrogel [24]. Bone and teeth of most animals, including humans, are composed of calcium phosphate (e.g., HA) which makes up 62–65% of the total bone composition [25]. Calcium phosphates have intrinsic properties that stimulate bone regeneration [26,27,28]. Therefore, presence of calcium phosphates, such as HA, is expected to improve the bioactivity of the designed PCL-gel samples [28,29,30]. Alginate is a hydrophilic anionic polysaccharide and exhibits chelation in the presence of divalent cations such as Ca^2+^ and Mg^2+^ [31]. Since alginate is unable to interact with cells, gelatin was added to improve cell adhesion [32,33].

Since hydrogels are mechanically fragile, to improve its retention at the application site, the hydrogel was loaded in a 3D printed gyroid PCL and post-crosslinked. Furthermore, a gyroid structure is characterized as having a minimal surface area and architectural as well as mechanical characteristics similar to trabecular bone [34,35,36]. The highest amount of hydrogel loading capacity in the gyroid scaffolds could be related to the larger pore size and minimal surface area as compared to other scaffold designs [35]. This network of larger sized channels can facilitate invasion of the host vasculature post in vivo implantation [37].

Apatite nucleation and growth is a dynamic process and depends on the concentration of calcium and phosphate ions in the SBF [28]. The entire process of apatite growth on biodegradable calcium phosphate-based biomaterials can be divided into two steps [38]. During the first step, the biomaterial (hydrogel) dissolved and supplemented the SBF with calcium and phosphate ions until super-saturation was achieved [39]. In the second step, calcium and phosphate ions started depositing on the biomaterial surface from the supersaturated solution [39]. In the case of PCL, decline in the amounts of apatite could be correlated to the bioinertness of PCL [40]. In contrast to this, an increase in apatite on the hydrogel with time was due to the bioactive nature of the hydrogel [41]. It is known that a dissolution study in SBF can simulate in vivo physiological conditions allowing dynamic interplay of material dissolution accompanied by bone mineralization and deposition [42]. Since the designed PCL-gel system is bioactive, it can support the osteogenic differentiation of hMSC environment [43,44] and thus, can accelerate osseointegration [45] when applied in vivo.

Although the primary objective of this study was to develop a bioactive and biodegradable hybrid system of a polymeric scaffold and hydrogel, we also aimed to create an easy and rapid system for on-demand applications in bone tissue engineering. A custom-made 3D printed reconstruct implant specifically designed to fit in the defect can minimize the micromotion of the implant at the host site and enable firmly-anchored new bone formation at the interface of implant and bone. In addition, a rapid method for fabrication of a 3D printed scaffold–hydrogel hybrid system can be used to reconstruct and stabilize architecturally complex bone fractures [11].

In summary, the designed hybrid system of a PCL scaffold and bioactive hydrogel was cytocompatible with an ability to promote apatite formation. Therefore, the designed PCL-gel system can potentially be used to repair custom-sized bone defects. However, further studies are required to test the efficacy of this system for promotion of vascularization and osseointegration.

## 4. Conclusions

The hybrid PCL-gel system indicated good cytocompatibility, showing adhesion and viability of the hMSC within the hydrogel matrix as well as on the solid scaffold surfaces. Further, the biomineralization test in SBF showed the nucleation and growth of apatite crystals on the hydrogel as well as the PCL scaffold, which confirmed its bioactivity. This hybrid PCL-gel system can be optimized to fabricate an implantable device within a short time to provide on-demand patient-specific solutions. Overall, the present study provides a new approach in bone tissue engineering for repair of bone defects, with a bioactive hybrid system of a biodegradable scaffold and hydrogel. Furthermore, in vitro studies could be carried out in future to combine endothelial cells and growth factors in addition to hMSC to induce vascularization. Also, in vivo studies could be carried out to study the effect of dissolution rate of the implant and new bone formation on the overall bone repair process.

## 5. Materials and Methods

### 5.1. Materials

Sodium alginate (Cat. No. 218295) and type-A gelatin (Cat. No. 901771) was obtained from MP Biomedicals (Strasbourg, France). 1-ethyl-3-(-3-dimethylaminopropyl) carbodiimide hydrochloride (EDC, Cat. No. 22980) and *N*-hydroxysuccinimide (NHS, Cat. No. 24500) were purchased from Thermo Scientific (Rockford, IL, USA). Calcium chloride (Cat. No. C79-500) was purchased from Fisher Chemicals (Fair Lawn, NJ, USA). Nanocrystalline HA was synthesized using a previously reported method of suspension-precipitation with calcium oxide and orthophosphoric acid added as a precursor [46]. HA powder was prepared during one of our previous study [47] and was used in this study without any modification. HA particles were characterized by length and width of 120 ± 38 nm and 52 ± 25 nm, respectively [47]. PCL (Cat. No. B01M8IDB07) filament with 1.75 mm diameter was obtained from Shenzhen Esun Industrial Co. (Shenzhen, China). In addition, hMSC (Cat. No. SV30110.01), basal culture medium (Cat. No. SH30879.01), and growth supplement (Cat. No. SH30878.01) were obtained from HyClone Laboratories, GE Healthcare Life Sciences (Logan, UT, USA). A green florescent dye (PKH67, Cat. No. MINI67) for the pre-staining of cells prior to cell culture was purchased from the Sigma Aldrich (St. Louis, MO, USA). 1× Cell Dissociation Medium (0.25%Trypsin supplemented with 2.21 mM EDTA, Cat. No. 25-053-Cl) and 1× Phosphate Buffered Saline (1× PBS, Cat. No. K812-500) were purchased from Mediatech, Corning (Masassas, VA, USA) and Amresco (Solon, OH, USA), respectively. A sterilized syringe of 10 mL volume (Cat. No. DG515805) and a needle of 0.7 mm inner diameter (Cat. No. B01KZ0MHSC) were procured from Becton Dickinson (Franklin lakes, NJ, USA) and Huaha (China), respectively. For the bioactivity study, 1× simulated body fluid (1× SBF) was prepared according to the method described by Oyane et al. [48] and has been reported elsewhere [28].

### 5.2. 3D Printing of PCL Scaffold

In this study, cylindrical-shaped (height: 5 mm, diameter: 15 mm) PCL scaffolds of gyroid structure (65% porosity and 1.2 mm pore size) were printed using a Fused Deposition Modeling (FDM) printer (MakerBot, New York City, NY, USA, Model. Replicator Mini 5th generation) (Figure S3). Printing was accomplished with a nozzle diameter of 0.4 mm, operating temperature of 110 °C, and a printing speed of 90 mm/s with 100% material filling density. Prior to use, the scaffolds were washed in distilled water and sterilized by soaking in 70% ethanol for 20 min, followed by UV irradiation for 30 min. The extent of porosity of these scaffolds was measured by comparing the weight of porous scaffolds with solid scaffolds of similar dimensions.

### 5.3. Synthesis of Bioactive Hydrogel Infiltrated with hMSC

The method used for hydrogel synthesis was adopted from previous studies carried out by Wang et al. [49]. Briefly, 50 mg nanocrystalline powder of HA was added in 10 mL distilled water and mixed by magnetic stirring (at 100 rpm for 15 min at room temperature). Next, gelatin and sodium alginate, 200 mg of each were added and stirred again (at room temperature for 15 min at 100 rpm). After this, 25 mg EDC was added (stirred at room temperature for 10 min), followed by addition of 15 mg NHS (stirring for another 5 min at room temperature) to make the hydrogel mixture. For sterilization, this hydrogel was irradiated with UV light for 30 min. After sterilization, hMSC were mixed with the hydrogel. For doing this, first, hMSC were cultured in a T-75 flask, until ~70% confluency was reached. ~70% confluent layer of hMSC was trypsinized using trypsin-EDTA and cells (cell density ~2 × 10^7^ cells/mL) were pre-stained with PKH67 as per manufacturer’s protocols. These pre-stained cells were centrifuged to remove the cell-suspension media and mixed with hydrogel. Hydrogel mixed with hMSC was loaded in a sterilized syringe and was injected in the pores of scaffolds. A detailed method of hydrogel loading in the scaffold is provided in the following Section 5.4.

### 5.4. Formation of a Hybrid PCL/Hydrogel System

The cell-loaded hydrogel was filled in a sterilized syringe and injected in the 3D printed porous PCL gyroid scaffold. This “hybrid PCL/hydrogel system” was addressed as “PCL-gel sample” thereafter, was treated with 1 M CaCl_2_ for 10 min to prevent the leakage of hydrogel from the scaffold, followed by washing with 1× PBS for 10 min. Furthermore, PCL-gel samples were washed twice for 5 min each using complete culture medium (basal medium with 10% growth supplement). These PCL-gel samples with hMSC were then transferred to 24 well-plate and incubated in the presence of complete culture medium (5% CO_2_ and 95% relative humidity at 37 °C). The details of cell culture and viability analysis are reported in Section 5.5.

The gyroid structure was compared with commonly used mesh and honeycomb structures for its efficacy to allow the high loading of hydrogel within the pores of scaffolds. For this, the weight of 3D printed scaffolds of identical size and shape was measured before and after the hydrogel (without cells) loading in the scaffolds. The difference in weight was used to estimate the amount of hydrogel loaded in the scaffolds using Equation (1). In addition to this, PCL-gel samples without cells were used for the dissolution study and bioactivity test as well.

Amount of loaded hydrogel in the scaffold (mg) = *m*_h_ − *m* (1)

where, *m*_h_ and *m* was weight of hydrogel loaded scaffold and bare scaffold, respectively.

### 5.5. Cytocompatibility Assessment

PCL-gel samples (with pre-stained hMSC in hydrogel) were transferred to a 24 well-plate and incubated in the presence of 2 mL complete culture medium for 48 h (in 5% CO_2_ and 95% relative humidity at 37 °C). After 48 h of incubation, samples were observed under the fluorescent microscope to investigate cell adhesion, growth, and viability. At least 3 samples were used the study.

### 5.6. Dissolution Study and Bioactivity Test

To analyze the dissolution behavior and bioactivity, PCL-gel samples were immersed in 2 mL simulated body fluid (1× SBF) in 24 well-plate and incubated for 3, 6, and 12 days. Samples were incubated at 37 °C, 5% CO_2_ and 95% relative humidity, and media was not changed during the incubation period. At least 3 samples were used in each category.

#### 5.6.1. Calculation of the Dissolved Amount of Hydrogel

After completion of incubation, PCL-gel samples were carefully removed from the media and stored at −80 °C for 12 h in a closed container for SEM analysis. The detailed method of analysis is provided in the consecutive Section 5.7.

After removal of PCL-gel samples, SBF solution was transferred to wells of a 96 well-plate to record the absorption (optical density) at 630 nm using optical density reader (Model: EL*x*800, BioTek, Winooski, VT, USA) and data was compared with the optical density (OD) of untreated 1× SBF, used as a reference during absorption measurements.

To calculate the weight of dissolved hydrogel in SBF during incubation, first a standard curve was plotted using known amount of hydrogel, dissolved in 1× SBF (0.5, 1, 1.5 mg/mL) and corresponding OD (at 630 nm) was recorded (Figure S4). The Equation (2) obtained from the standard curve was used to estimate the amount of dissolved hydrogel in the SBF.

(2) $$Dissolved\;amount\;of\;hydrogel\;\left(\frac{\mathrm{mg}}{\mathrm{mL}}\right)=\;\left(\frac{0.0003+OD_{s}}{0.0157}\right)$$

where, *OD*_s_ was the optical density of the SBF after 3, 6, and 12 days of immersion testing.

#### 5.6.2. Apatite Formation on the PCL-Gel Samples

As mentioned in the previous section, after dissolution study, PCL-gel samples were removed from the SBF and refrigerated for 12 h at −80 °C. These refrigerated samples were then lyophilized for 12 h and after this, were analyzed using scanning electron microscope (SEM, S-4800, Hitachi, Tokyo, Japan) to study the morphology of deposited apatite on samples as well as the mechanism of apatite formation. Apatite formation data was correlated with the dissolution data to better understand the relationship between dissolution and deposition.

### 5.7. XRD and SEM Analysis

For the phase analysis, PCL-gel samples without cells were lyophilized prior to X-ray diffraction (XRD, D8 Discover, Bruker’s diffractometer, Karlsruhe, Germany). XRD was carried out at 40 kV voltage and 40 mA current with CuKα wavelength (1.54056 Å) and 2θ ranges from 20° to 90° at a scanning rate of 2°/min with a step size of 0.02°.

SEM was operated in secondary electron mode for the analysis of morphology of PCL-gel samples, before and after dissolution study. Scaffolds without hydrogel were also studied and compared with the PCL-gel samples. Prior to SEM, to minimize charging during observation, samples were gold-coated using sputter coater (Model: EMS150R ES, Quorum, Laughton, East Sussex, UK), equipped with a gold/palladium target (Cat. No. 91017-AP, Electron Microscopy Sciences, Hatfield, PA, USA).

## Supplementary Materials

The following are available online at http://www.mdpi.com/2310-2861/3/3/26/s1, Figure S1: Digital pictures of the pre-hydrogel prepared for loading in the 3D printed PCL scaffolds; Figure S2: Reaction process of hydrogel synthesis involved in this study. This reaction involved the activation of carboxyl groups of alginate by EDC to form active ester groups, followed by replacement of EDC by NHS to improve the efficiency of amine reaction. Afterward, NHS in NHS activated carboxylic group of alginate was replaced by primary amine of gelatin. Finally, CaCl_2_ addition led to the ionic interactions of α-L-guluronic acid (G-block) of alginate to form hydrogel; Figure S3: STL image of gyroid-shaped three-dimensional cylindrical scaffold (a), side (b), and top (c) view of 3D printed gyroid scaffold of PCL, and SEM images of the scaffold to show the porous morphology (d); Figure S4: Standard curve, plotted between amounts of dissolved hydrogel and measured optical density at 630 nm; Figure S5: Representative SEM images of apatite grown on hydrogel (in PCL/hydrogel scaffold) after 12 days of immersion in SBF. The crack generated due to drying-induced strain was used to estimate the apatite layer thickness; Figure S6: Representative bright field (a) and fluorescence with 488 excitation laser (b) confocal microscopy images of hydrogel-loaded PCL scaffold without pre-stained cells to study the auto fluorescence of hydrogel, nano-hydroxyapatite and PCL scaffold. Results confirmed no auto fluorescence from hydrogel, nano-hydroxyapatite and PCL scaffold.
