# Supplementary material for: A Bioactive Hydrogel and 3D Printed Polycaprolactone System for Bone Tissue Engineering
Source: Gels. 2017 Jul 6;3(3):26. doi: 10.3390/gels3030026 (PMC5770986; doi:10.3390/gels3030026)
Supplement: Supplementary file 1 [file gels-03-00026-s001.pdf]

Supplementary Information

A Bioactive Hydrogel and 3D Printed Polycaprolactone System for Bone Tissue Engineering

Ivan Hernandez, Alok Kumar and Binata Joddar

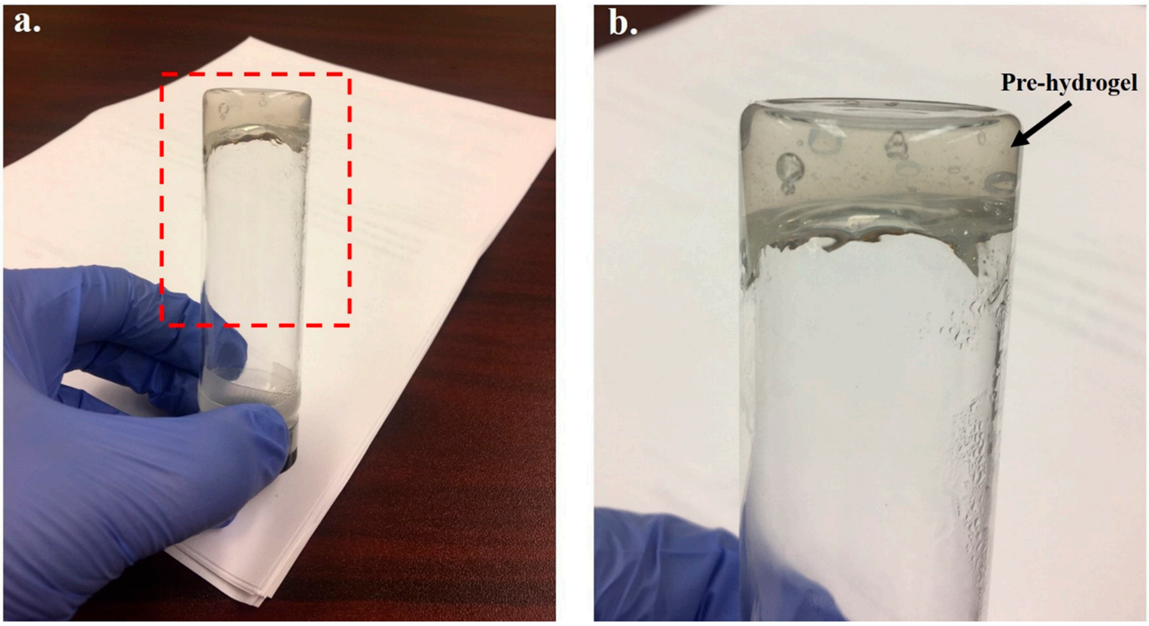

Figure S1. Digital pictures of the pre-hydrogel prepared for loading in the 3D printed PCL scaffolds.

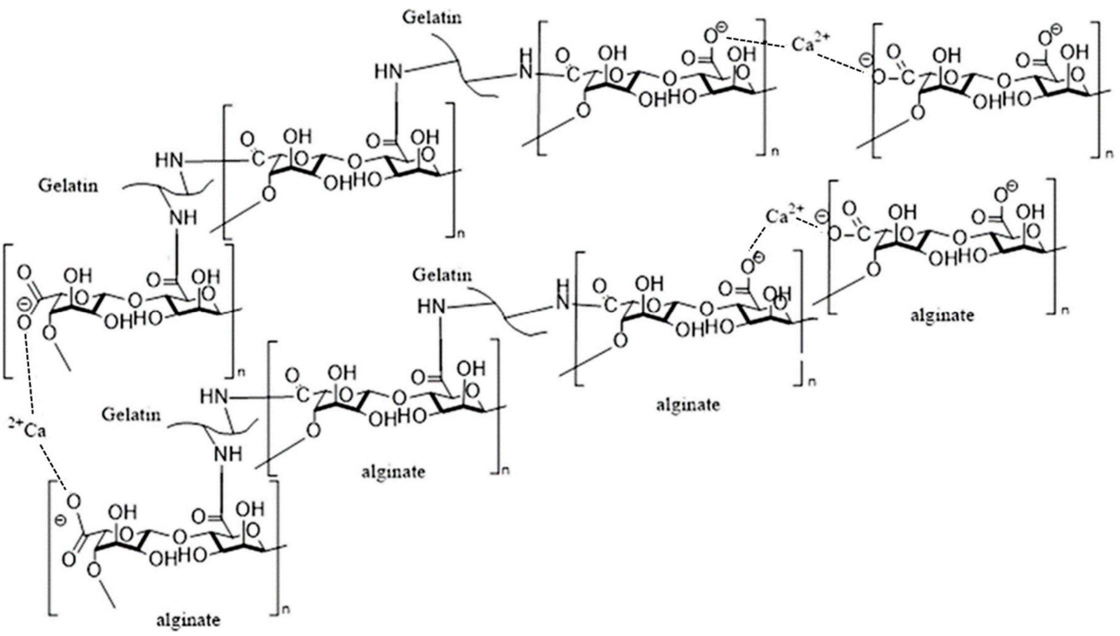

Figure S2. Reaction process of hydrogel synthesis involved in this study. This reaction involved the activation of carboxyl groups of alginate by EDC to form active ester groups, followed by replacement of EDC by NHS to improve the efficiency of amine reaction. Afterward, NHS in NHS activated carboxylic group of alginate was replaced by primary amine of gelatin. Finally, $CaCl_2$ addition led to the covalent linkage of α-L-guluronic acid (G-block) of alginate to form hydrogel.

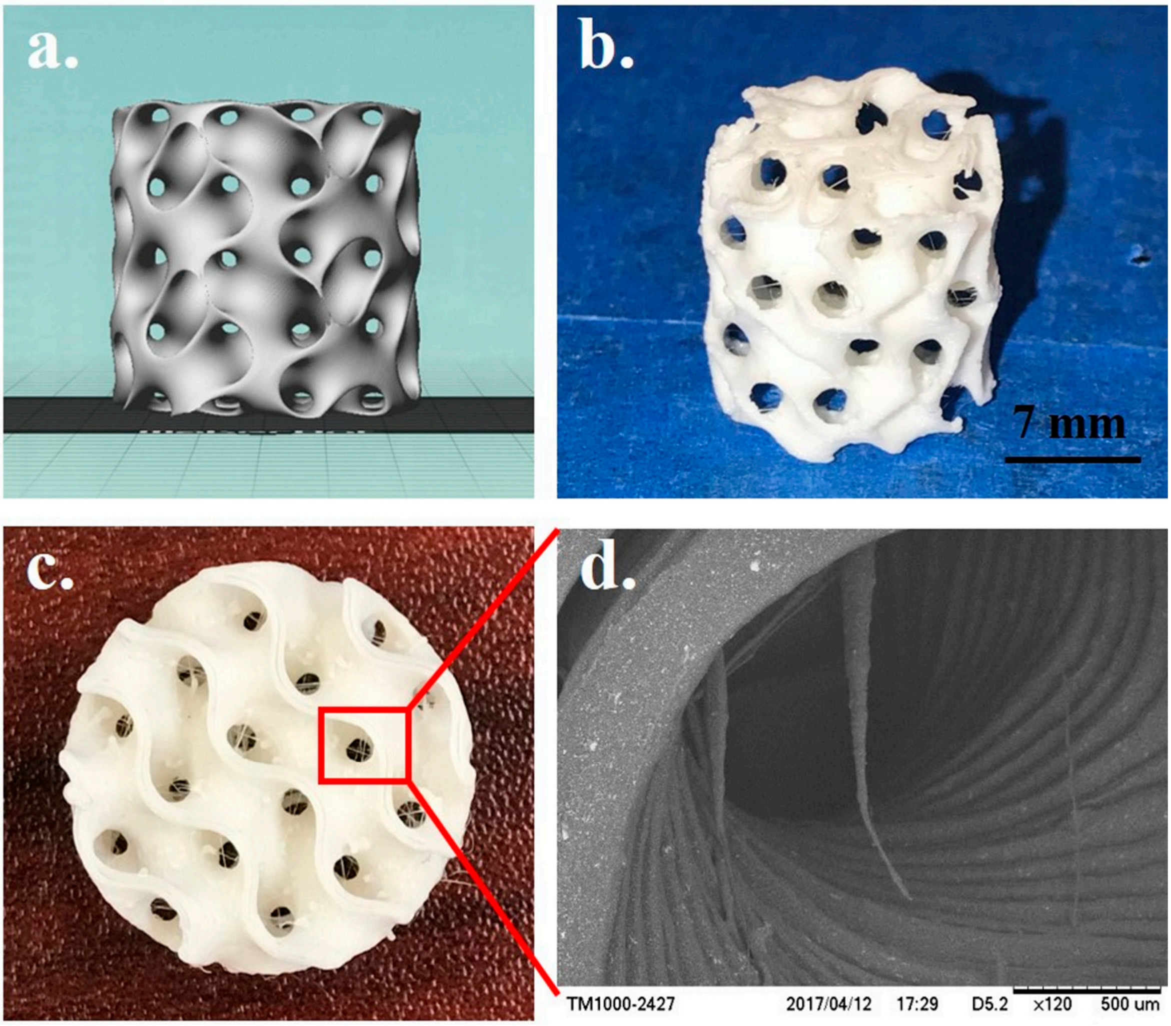

Figure S3. STL image of gyroid-shaped three-dimensional cylindrical scaffold (**a**), side (**b**), and top (**c**) view of 3D printed gyroid scaffold of PCL, and SEM images of the scaffold to show the porous morphology (**d**).

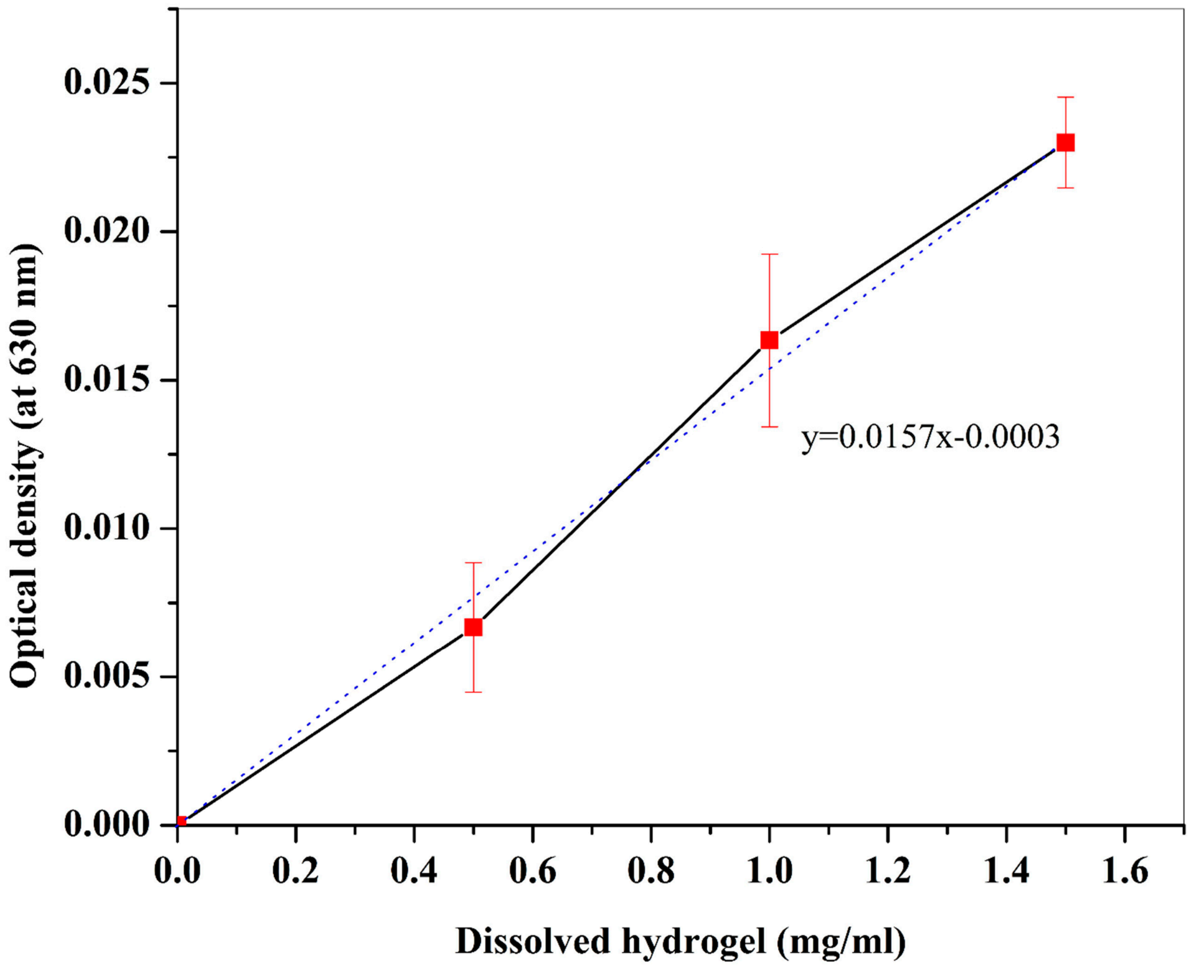

Figure S4. Standard curve, plotted between amounts of dissolved hydrogel and measured optical density at 630 nm.

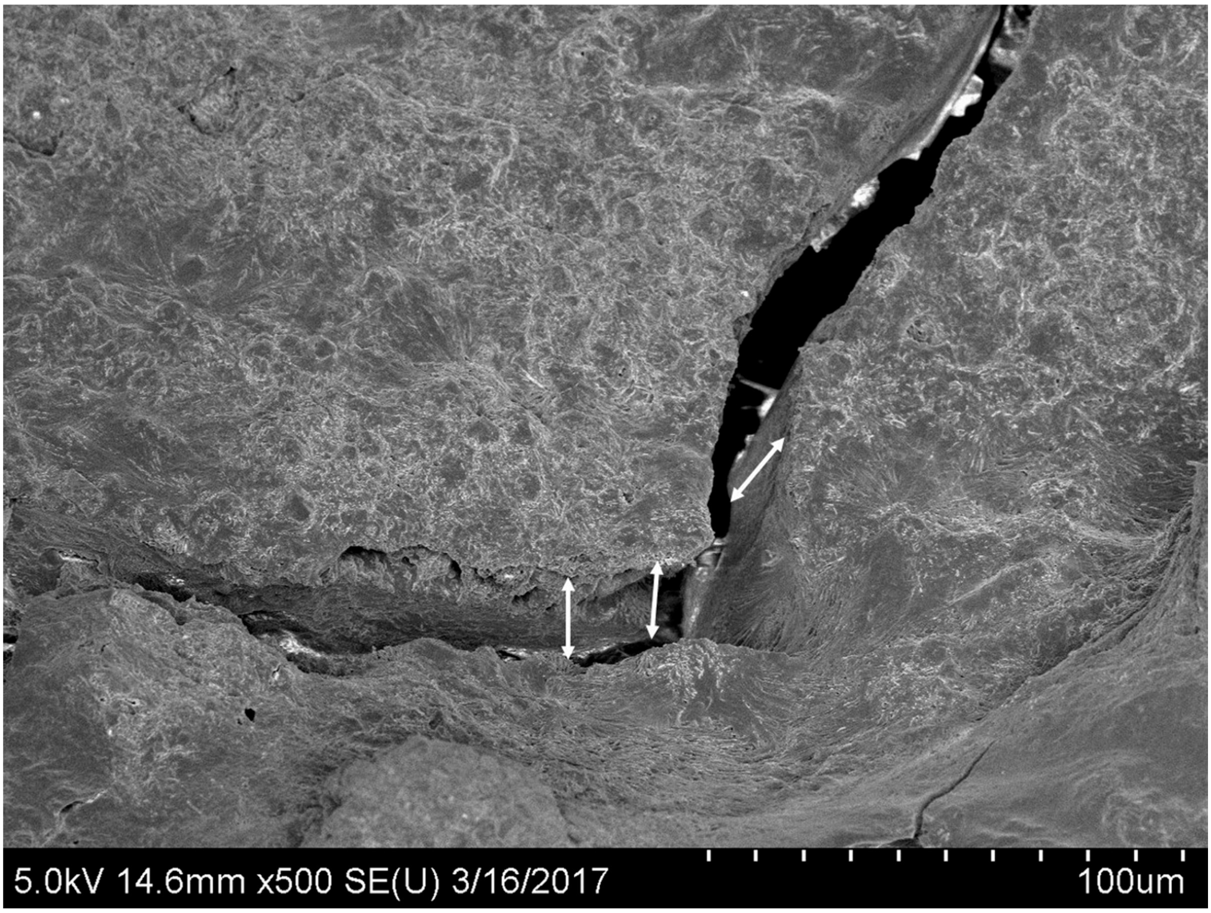

Figure S5. Representative SEM images of apatite grown on hydrogel (in PCL/hydrogel scaffold) after 12 days of immersion in SBF. The crack generated due to drying-induced strain was used to estimate the apatite layer thickness.

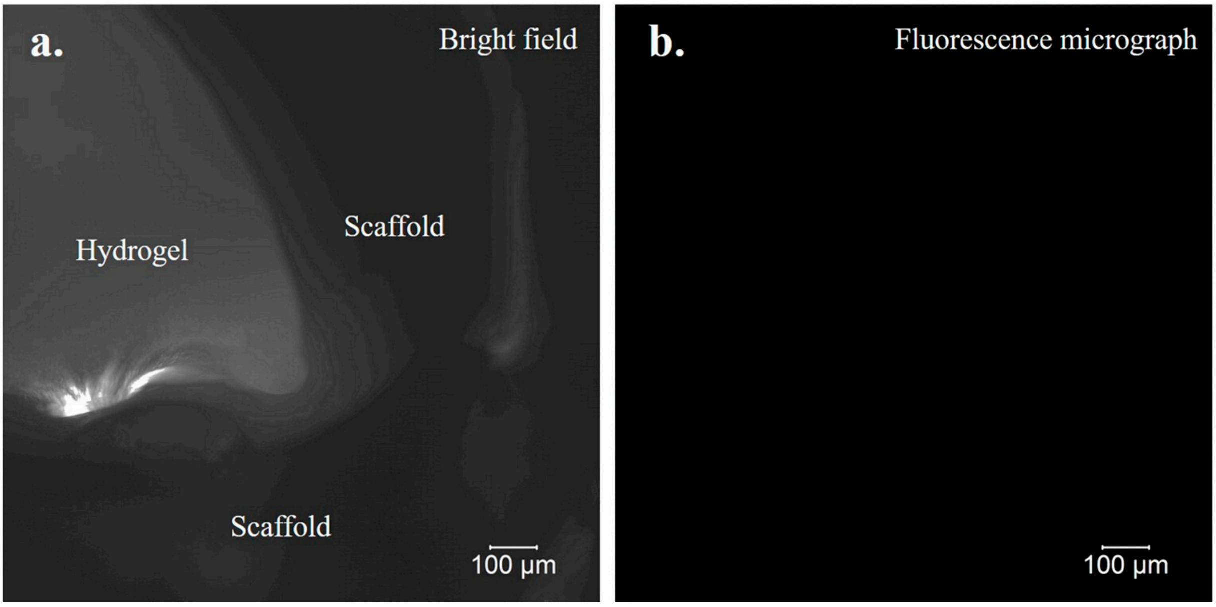

Figure S6. Representative bright field (**a**) and fluorescence with 488 excitation laser (**b**) confocal microscopy images of hydrogel-loaded PCL scaffold without pre-stained cells to study the auto fluorescence of hydrogel, nano-hydroxyapatite and PCL scaffold. Results confirmed no auto fluorescence from hydrogel, nano-hydroxyapatite and PCL scaffold.
